# Landfill Leachate Treatment by Using Second-Hand Reverse Osmosis Membranes: Long-Term Case Study in a Full-Scale Operating Facility

**DOI:** 10.3390/membranes12111170

**Published:** 2022-11-21

**Authors:** Raquel García-Pacheco, Albert Galizia, Sergi Toribio, Jordi Gabarró, Serena Molina, Junkal Landaburu-Aguirre, Francisco Molina, Gaetan Blandin, Hèctor Monclús, Ignasi Rodríguez-Roda, Joaquim Comas

**Affiliations:** 1 LEQUIA, Institute of the Environment, University of Girona, Carrer Maria Aurèlia Capmany 69, 17003 Girona, Spain; 2 IMDEA Water Institute, Avenida Punto Com, 2, 28805 Madrid, Spain; 3 TELWE S.A., Camprodon 49, 17240 Llagostera, Spain; 4 Grupo Sacyr, Sacyr Sadyt Industrial, Molina de Segura 8, 30007 Murcia, Spain; 5 Catalan Institute for Water Research (ICRA-CERCA), Emili Grahit 101, 17003 Girona, Spain

**Keywords:** landfill leachate, second-hand membranes, aged membranes, end-of-life, regeneration, reuse, reverse osmosis, circular-economy, sustainability, life span

## Abstract

Landfill leachate (LFL) has a complex inorganic, organic and microbiological composition. Although pressure-driven membrane technology contributes to reaching the discharge limits, the need for frequent membrane replacement (typically every 1–3 years) is an economical and environmental limitation. The goal of this work is to evaluate the feasibility of using second-hand reverse osmosis (RO) membranes to treat LFL in an industrially relevant environment. End-of-life RO membranes discarded from a seawater desalination plant were first tested with brackish water and directly reused or regenerated to fit with requirements for LFL treatment. A laboratory scale test of second-hand membrane reuse was carried out using ultrafiltered LFL. Then, a long-term test in an LFL full-scale facility was performed, where half of the membranes of the facility were replaced. The industrial plant was operated for 27 months with second-hand membranes. The permeate water quality fit the required standards and the process showed a trend of lower energy requirement (up to 12 bar lower transmembrane pressure and up to 9% higher recovery than the average of the previous 4 years). Direct reuse and membrane regeneration were successfully proven to be an alternative management to landfill disposal, boosting membranes towards the circular economy.

## 1. Introduction

Among the existing techniques for solid waste management, landfilling is the most widely used practice in the world [1,2]. According to Eurostat, from 2005–2020 period, the average municipal waste generation rate per capita in Europe was 505 kg/year [3]. The landfill leachate (LFL) is one of the by-products formed when landfilling. It is continuously generated through the degradation of organic matter and the percolation of natural rainfall [4,5]. Around 0.2 m^3^ of LFL is generated by each ton of solid waste [6], which needs to be drained, collected and effectively treated to avoid environmental damage [6]. 

The LFL general composition is very rich in a poorly biodegraded dissolved organic matter [2]. The composition and the ratio between 5 days biochemical oxygen demand (BOD5) and chemical oxygen demand (COD) changes over time (age). Besides, both are impacted by climate, hydrology and the composition of the municipal solid waste [7,8]. Since such contaminants are mostly not appropriate for treatment by conventional biological processes, new regulations tend to limit the discharge of such complex wastes to municipal sewers [9]. Due to the strict requirements for higher water quality in wastewater treatment and lower wastewater discharge (i.e., zero liquid discharge), the usage of advanced technologies is being imposed. Recently, new approaches have been reported in the literature, such as physical and chemical treatments for selective removal of heavy metals [10,11] or heterogeneous Fenton-like oxidation over copper-based catalyst organic removal [12]. However, currently, the process that combines a biological treatment method with advanced membrane treatment is one of the main approaches to treating mature LFL in the industry [13]. Mature LFL normally has a high content of refractory organic matter but also high alkalinity, high salinity, and high ammonia concentration [14]. 

The usage of membranes and particularly reverse osmosis (RO) have been recognized as one of the most efficient and economical ways to treat LFL, reducing its volume by 70 to 85% [9,15]. The treatment of LFL with RO started in the 1980s using tubular modules, spiral wounds and disc-tube modules [16]. Nowadays, spiral wound design is the most developed configuration [17], however, spiral-wound membranes have several drawbacks owing to their mechanism of separation and design. The volume reduction in LFL can reach up to 95% when combining RO and nanofiltration (NF) membranes under specific conditions [9]. 

On the other hand, the concentrate stream has to be handled. Several possibilities are listed. It can be: returned to the landfill by controlled reinjection [9,18,19], combined with controlled crystallization for further processing [9], evaporated and dried followed by deposition of the dry residues in a special landfill [9], transported to a specialist incineration plant [9], solidified/stabilized and disposal of the dry waste on the landfill itself [9], or coagulation [20]. The RO LFL permeate normally contains only very low levels of inorganic and organic contaminants and can be discharged into the environment [9] or can be reused.

Compared to seawater (SW) desalination facilities, LFL plants host a relatively small number of membranes (generally <200 RO modules). On-site treatment costs using RO membranes range from 15€·m^−3^ to 40€·m^−3^ [1]. However, the complexity of the LFL composition limits the spiral-wound RO membrane lifespan up to 3 years. The need of frequent replacement is an economical and environmental issue concerning industry, which normally pays between 500€ and 1000€ for a single spiral wound RO module (data based on interviews of membrane operators), and generally disposes of the end-of-life membranes in landfills [21]. In fact, by 2025, around two million end-of-life RO membranes per year will be discarded from industrial processes (i.e., desalination) [22]. Indiscriminately, membrane landfill disposal is relatively cheap (less than 10€/end-of-life membrane [21]), but is against sustainability and circular economy. Although, in the last two decades many studies have been pointing out the possibility of RO membrane recycling and reuse, as summarized on the REMapp website [23], so far most of the attempts have been only focused at laboratory [24,25,26,27] or pilot scale [28,29,30]. 

The goal of the present work is to demonstrate the reuse of second-hand membranes for treating LFL in long-term and full-scale operations. In order to reach the main objective, the following approach was followed: (i) end-of-life RO membrane sorting (brackish water (BW) pilot test) and regeneration, (ii) reusing of second-hand membranes to treat LFL at laboratory scale and, (iii) reusing of second-hand membranes for LFL treatment at full scale and long-term operating conditions. In the latest, half of the system was operated with standard seawater reverse osmosis (SWRO) membranes, and the other half with second-hand SWRO membranes (both directly reused and regenerated). Follow-up of the permeate water quality and operating conditions was conducted up to 27 months and values were compared with the previous 4 years of operation data (2016–2019) using standard SWRO membranes. This study demonstrated the feasibility of giving a second life for the end-of-life SWRO membranes at an industrial scale and will help to transfer to the market such circular economy practice.

## 2. Materials and Methods

### 2.1. End-of-Life Membranes

All assays were performed on spiral wound end-of-life polyamide (PA) RO membranes (8″ diameter modules) from LG and Toray previously used for SW desalination (in Spain). Table 1 shows all the membranes used in this study, indicating: (i) internal code, (ii) membrane model, (iii) physical damage, and (iv) what they were used for within this study (i.e., membrane sorting, regeneration, reuse test at lab scale, reuse test at industry and autopsy). All membranes were first tested in a pilot plant using natural BW. As a function of their low permeate flux, 5 out of 12 membranes were regenerated to improve their final performance. Finally, 3 out of the 7 remaining end-of-life membranes were directly reused for industrial LFL treatment. Laboratory tests and autopsies were conducted on 4 of them.

From now on, end-of-life RO membrane terminology is used to indicate membranes which were directly reused. Regenerated membranes terminology is used for membranes that were treated according to Section 2.3 and further reused in this study. In both cases, membranes are called second-hand membranes due to the prolongation of their first lifespan.

### 2.2. End-of-Life Membrane Sorting

Membranes were weighed after being drained for 1 h. Then, they were characterized using natural pre-filtered (sand filtration) BW from Cuevas del Almanzora desalination plant managed by Sacyr Sadyt. A pilot-scale cross-flow system described elsewhere [30] was used. REMapp road map decision-making tree [23] was used to decide the fate of the collected end-of-life RO membranes in terms of being (i) reused as RO, (ii) regenerated as RO, (iii) recycled as NF, (iv) recycled as ultrafiltration (UF) or (v) discharged to incineration or landfill [23]. In this study, membranes with external damage were discarded from industrial testing and reused only for laboratory tests (see Section 2.4) and autopsy (Section 2.9). Once the end-of-life RO membranes were sorted, the regeneration protocol was applied to 5 out of the 12 membranes and the regenerated membrane performance was tested again. The operating conditions and BW characteristics are shown in Table 2.

### 2.3. Regeneration Membrane Process

The applied protocol for membrane regeneration was based on García-Pacheco et al. [30], which uses a passive reactor containing 6 membrane housings. End-of-life RO membranes were exposed to a chemical solution with a controlled concentration, pH, and known exposure time to eliminate the organic fouling and improve the membrane performance. The solution was prepared and transferred to the reactor. 5 out of 6 regenerated membranes were refurbished at pilot scale, maintaining their fiber-glass casting. Only membrane M1 was regenerated at laboratory scale. It showed physical external damage and therefore was discarded for further industry implementation but still used for further laboratory assessment (Section 2.4). In order to have an end-of-life RO membrane sample, an autopsy was performed on membrane M1. Several coupons were kept in end-of-life state and other coupons were regenerated at the same conditions as applied in the pilot reactor. Both end-of-life and regenerated coupons from M1 were further used in the laboratory for LFL filtering tests.

### 2.4. Reuse of Second-Hand Membranes to Treat Landfill Leachate: Laboratory Tests

A laboratory-scale cross-flow test system was used to evaluate three flat sheet membrane coupons performance: one end-of-life RO (M1) and two regenerated RO (M1 and M10). Thus, M1 and M10 were autopsied, and flat coupons were extracted (see Section 2.9). 

The main goal was to assess if the permeate water production and quality fit the industrial requirements, before installing the 8-inch diameter spiral-wound RO membranes in the LFL industrial facility. Therefore, ultrafiltered LFL was collected from the industrial facility and used as feed water. The water quality of the ultrafiltered LFL used as feed is shown in Table 3. The filtering system, with an effective membrane filtration area of 84 cm^2^ is described in García-Pacheco et al. [24]. The system was operated at 45 bar transmembrane pressure (TMP) at room temperature for 1 h before the collection of permeate for water quality analyses, ensuring stable performance. 

### 2.5. Reuse of Second-Hand Membranes in a Full-Scale Landfill Leachate Facility: Long-Term Test

AN LFL facility managed by TELWESA (located in Catalonia) was selected to assess the application of second-hand membranes for treating LFL for 27 months, under real operating conditions.

The landfill where the LFL treatment plant operated is used to dispose of non-hazardous waste. The LFL treatment plant features a pressurized external cross-flow membrane bioreactor from Wehrle-Werk (Biomembrat^®^) that allows nitrogen removal by nitrification-denitrification (working at up to 40 °C). The UF permeate is collected in a reservoir. Depending on the water quality and the downstream water usage, the UF permeate is either filtered by activated carbon or sent to RO process. Sulphuric acid and antiscalant are added prior to the RO process for scaling prevention (pH 6). The maximum treatment capacity of the plant is 50 m^3^·day^−1^.

**Figure 1:**
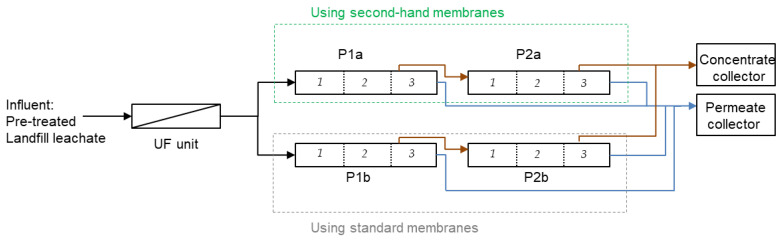
RO LFL treatment plant with one unique step.

The RO unit is composed of 12 RO membrane modules (8-inch diameter, SWC5 LD-4040, Hydranautics). The system has 4 pressure vessels (Figure 1) arranged in one step with feed water flowing from the bottom (P1a and P1b pressure vessels) to the top (P2a and P2b pressure vessels). Feed flow, permeate flow, water temperature, and pressure are continuously controlled, and automatically collected using a data logger. There is a permeate collector sampler in each pressure vessel, while there is only a common sampler in the feed and concentrate side. Typically, before installing the second-hand membranes (historic data 2016–2019) the plant operated at 47 ± 5 bar treating 1.2 ± 0.2 m^3^·h^−1^ with a total conversion of 62 ± 9 %. The feed water quality entering the RO unit is shown in Table 4.

Second-hand membranes were installed according to Table 5. Second-hand membranes were installed in 2 out of 4 pressure vessels of the industrial facility. End-of-life membranes were installed in the pressure vessel P1a and the regenerated membranes in the pressure vessel P2a. In each case, the position of the membranes was selected according to the permeate flux shown in the preliminary filtering test (see Section 2.2). The membranes with the lowest permeate flux were placed on the feed side (position 1), while the membranes with the highest permeate flux were placed on the concentrate side (position 3). The day before the membrane substitution, alkaline standard chemical cleaning was applied for 6 h in order to reduce the amount of fouling on the standard RO membranes of the facility.

### 2.6. Membrane Performance

In all the systems, pilot (BW pilot test for membrane sorting), laboratory scale (LFL) and full-scale (LFL), the permeation flux (*J*, L·m^−2^·h^−1^), was calculated following the Equation (1), where *Q_p_* (L·h^−1^) is the permeate water flow, *S* (m^2^) is the active membrane surface area. The *TMP* (bar) was estimated as the average between the inlet (*P_f_*) and outlet (*P_c_*) feed channel pressure (Equation (2)). In the case of the pilot and the laboratory systems, atmospheric pressure (zero manometric bar) at the permeate side (*P_p_*) was assumed. The rejection coefficients (%R) were calculated using Equation (3), where *C_f_* and *C_p_* are the salt concentration found in the feed and permeate, respectively. The percentage of recovery (%*Recovery*) was calculated by dividing the permeate flow (*Q_p_*) by the feed flow (*Q_f_*) (Equation (4))

(1) $$J=\frac{Qp}{S}$$

(2) $$TMP=\frac{P_{f^{}}+P_{c^{}}}{2}-P_{p}$$

(3) $$\%R=\left(1-\frac{C_{p}}{C_{f}}\right)$$

(4) $$\%Recovery=\frac{Qp}{Qf}\cdot 100$$

### 2.7. Water Quality Analyses 

Different equipment was used according to the water quality of the samples to determine the individual ion and organic compounds rejection. The results were introduced into Equation (3), where *C_p_* and *C_f_* now represent the concentrations of the corresponding analyses.

#### 2.7.1. Water Quality Analyses at BW Pilot Test for Membrane Sorting and Laboratory Scale Test

Ion compounds were measured by ion chromatography (IC) using an 861 advanced compact Metrohm ionic chromatograph with an autosampler 838 Advanced Sample Processor. Organic compounds rejection coefficients were determined by measuring the total organic carbon (TOC) concentration using a TOC-V CSH Shimadzu device (UNE-EN 1484:1998).

#### 2.7.2. Water Quality Analyses at the Full-Scale Industrial Facility

Historic data of water quality provided by TELWESA were obtained by in situ measurement using pH-meter, conductivity-meter, and spectrophotometry analysis for COD, nitrate and ammonia (i.e., LCK514 and LCK314 (COD), LCK340 (Nitrate) and LCK 302 and LCK303 (ammonia), Hach kits) and chloride ions (Aquamerck 200, Merk). During this study and due to the complexity of LFL water quality, only the permeate samples of the four pressure vessels were fully characterized as mentioned in Section 2.7.1. Besides, BOD_5_ was analyzed using a respirometric sensor system (APHA, 5210 B). 

### 2.8. Operational Data Analysis of Landfill Leachate Treatment at the Full-Scale Industrial Facility

Operation data were statistically analyzed using R to check differences between 4 different operational periods ranged from 2019 to 2022. The first period corresponds to the conventional operation with commercial membranes (0% of the system was using second-hand membranes). The second period corresponds to the first membrane replacement (P1a pressure vessel, month 0), were 25% of the system was operated with second-hand membranes. The third period corresponds to the second membrane replacement (P2a pressure vessel, month 1), were 50% of system was operated with second-hand membranes. Lately, the fourth period corresponds with the last membrane replacement (month 10), where again, only 25% of the system was operated with second-hand membranes.

The percentage of recovery (Equation (4)) was selected to compare these operational periods. The normality of the data and variances comparison was done through Kolmogorov-Smirnov test and F-test respectively. Then, the statistical differences between mean values of each period were also compared. 

### 2.9. Membrane Autopsy

Membranes with physical external damage such as M1 (LGSW440GR model) and M10 (TORAY TM820M 400) were chosen for the laboratory analysis. To conduct the laboratory analysis, a membrane autopsy is required to extract the membrane coupons to fit in the lab scale filtering system. Consequently, the destruction of the module was required as shown elsewhere [31].

However, further studies of fouling and surface characterization were only conducted for LGSW440GR model, which is the one used for the long-term study. An autopsy was performed on 3 LGSW440GR membrane modules to study the membrane surface before and after being regenerated (M1) and to characterize the fouling deposition on the membrane surface after treating LFL in the industrial facility (M4 and M5).

The membrane surface morphology beore and after applying the regenerating treatment (M1) was characterized by Scanning Electron microscopy (SEM) using a S-8000 Model (Hitachi) image device. Additionally, membrane surface was characterized by attenuated total reflectance-Fourier transform infrared (ATR-FTIR) spectroscopy using a Perkin-Elmer RX1 spectrometer, equipped with an internal reflection element of a diamond at an incident angle of 45°. An adequate pressure was applied to the membrane placed on the crystal surface. The spectra were recorded at a resolution of 2.0 cm^−1^ in the frequency region of 4000–650 cm^−1^, with an average of 4 scans per sample. Previously the samples were dried at 100 °C for two days. ATR-FTIR spectroscopy was used to determine if the PA layer was impacted by the regeneration treatment.

Fouling samples were also collected from the second-hand membranes used to treat LFL at the industrial facility (M4 and M5) and dried at 105 °C to determine the quantity of fouling deposition per membrane surface (dry weight expressed in g·cm^−2^). Thermogravimetric analysis (TGA) was carried out to determine fouling nature (organic or inorganic). Analyses were carried out using a Mettler Toledo TGA/SDTA 851 instrument (Mettler-Toledo, Schwerzenbach, Switzerland). Fouling was heated from room temperature to 600 °C under an oxidative atmosphere at a 10 °C min^−1^ heating rate. Inductively coupled plasma mass spectrometry (ICP-MS) was used to assess the inorganic fouling composition. A spectrometer 7700× from Agilent Technologies (Santa Clara, California, USA) was employed. Accurately weighed fouling samples (50 mg) were digested with 4:1 ratio of trace metal analytical grade HNO_3_:H_2_O_2_ and then diluted in 100 mL of ultrapure water. Clear solutions were diluted and analyzed.

## 3. Results and Discussion

The results section is divided into three subsections: (i) end-of-life RO membrane sorting (BW pilot test) and regeneration, (ii) reuse of second-hand membranes to treat LFL at laboratory scale and, (iii) reuse of second-hand membranes for LFL treatment at full-scale and real operation conditions.

### 3.1. End-of-Life RO Membrane Sorting (BW Pilot Test), Regeneration and Recycling at Pilot Scale

#### 3.1.1. End-of-Life RO Membrane Sorting

**Figure 2:**
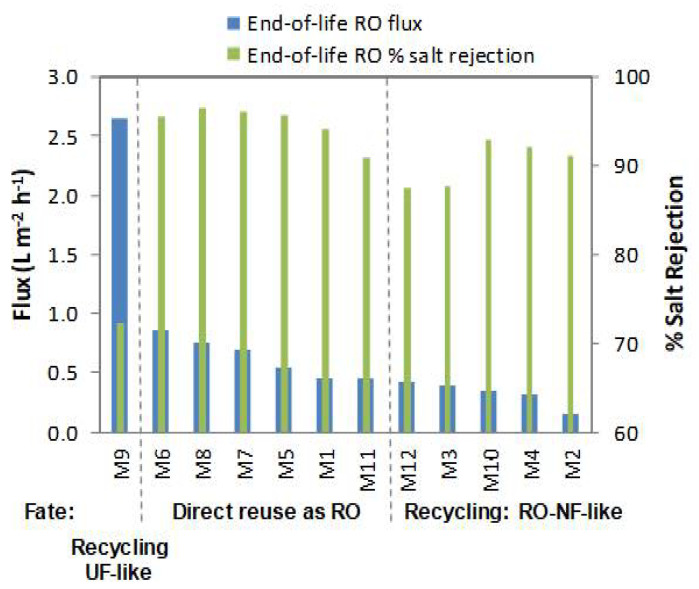
End-of-life RO membrane characterization to decide their management fate.

End-of-life RO membranes were characterized at 15.5 bar using natural BW as feed solution. Figure 2 shows flux and salt rejection (calculated by using conductivity data). All the end-of-life SWRO membranes weighed around 15 kg and had lower performance than new RO membranes (i.e., according to the manufacture datasheet at their test conditions. LG LGSW440GR: permeate flux 31.7 L·m^−2^·h^−1^ (permeability 0.57 L·m^−2^·h^−1^·bar^−1^) and salt rejection >99.7 %. Toray TM820M 400: permeate flux 29.8 L·m^−2^·h^−1^ (permeability 0.54 L·m^−2^·h^−1^·bar^−1^) and salt rejection >99.5 %). The flux of the end-of-life SWRO membranes ranged from 0.15 to 0.87 L m^−2^ h ^−1^ (0.01 to 0.06 L m^−2^ h ^−1^·bar^−1^) and the salt rejection ranged from 72.3% to 96.4%. The fate of the membranes was decided based on the combination of permeate flux values and rejection coefficients (Figure 2). Membranes that showed a permeate flux greater than 0.46 L·m^−2^·h^−1^ and have a rejection coefficient greater than 90.0% (membranes M5, M6, M7, M8, and M11) were selected to be directly reused without applying any further treatment. Membranes with a permeate flux lower than 0.46 L·m^−2^·h^−1^ (M2, M3, M4, M10, and M12 membranes) were regenerated to improve the permeate flux but maintain their separation capability. Membranes with higher permeate flux than 0.46 L·m^−2^·h^−1^ but low rejection capability (72.3%) such as the membrane M9 will be suitable for recycling into UF-like performance (not further used in this study). Membranes with physical external damage such as membrane M1 and M10 were chosen for the laboratory analysis; i.e., dismantled for autopsy (see Section 3.4).

#### 3.1.2. Regenerated Membrane Characterization: Permeability and Salt Rejection

**Figure 3:**
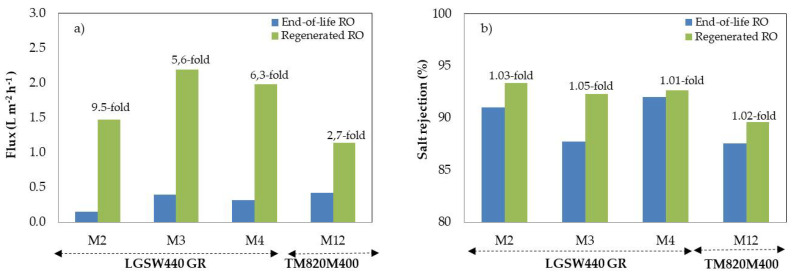
Flux (**a**) and rejection coefficients (**b**) of RO membranes before (end-of-life) and after (regenerated) being exposed to the regenerating solution.

Figure 3 shows end-of-life RO membrane fluxes and rejection coefficients before and after being exposed to the regenerating solution. After chemical exposure, membrane flux increased substantially, i.e., up to 9.5-fold (M2). Positively also, the rejection capability did not vary or slightly increased. On one hand, membrane permeability increases due to fouling elimination. On the other hand, once fouling is eliminated, the components of the regeneration solution could potentially interact with the highly reactive end amine groups of the PA RO layer, and the carboxylic group (R-COOH) could turn to (R-COO-) groups in the linear part of cross-linked aromatic PA [32,33]. Therefore, the hydrophilicity of the membrane surfaces increased leading to less resistance to water passage through the membrane [32]. Such phenomenon has been already observed in other studies [34,35].

### 3.2. Reusing of Second-Hand Membranes for Landfill Leachate Treatment: Laboratory Test

A preliminary laboratory filtration test was used to ensure the successful application of second-hand membranes in the full-scale LFL facility. Three membranes coupons: M1-end-of-life RO, M1-regenerated RO and M10-regenerated RO were used.

Table 6 shows permeate flux, permeability and the rejection coefficients when treating the pre-filtered LFL water, compared to the observed rejection at the industrial facility prior to installing any second-hand membranes (based on historic data 2016–2019 and a punctual sample of July 2019). 

The laboratory test confirmed the potential use of second-hand membranes for treating LFL in short-term operation, which showed greater water passage than the standard RO membranes. This could be attributed to the difference of filtering systems (lab scale vs industrial), but also the fact that the time used at lab scale was very short to foul the membrane coupons.

As it was observed in Section 3.1.2, the permeate flux of the regenerated M1 membrane (LGSW440GR) was around 1.5-fold higher than the end-of-life ones. Besides, the permeate flux of the regenerated membrane M10 (Toray TM820M-400) was 1.7-fold higher than the one of the regenerated membrane M1. Although the TM820M-40 model could have been a good candidate for further implementation at the industrial facility, it was discarded due to its rejection capability. Permeate samples of membrane M10 had a yellowish color, which could indicate low rejection for some low molecular weight organic compounds.

The second-hand membranes performed slightly better than the standard RO ones (Hydranautics SWC5 LD) installed in the full-scale facility in terms of: over salt rejection (measured by conductivity), and more specifically COD, BOD_5_, Cl^−^, N-NO_3_^−2^, N-NH_4_^+^ and SO_4_^−2^ ions rejections. Better performance of second-hand membranes than the standard RO membranes is highlighted in green in Table 6.

### 3.3. Reusing of Second-Hand Membranes in a Full Scale Landfill Leachate Facility

LGSW440GR membrane model was selected to validate the use of second-hand RO membranes in a full-scale LFL treatment facility accordingly to the laboratory scale results and stock availability.

End-of-life and regenerated RO membranes were installed in the LFL facility according to the permeate flux values obtained during their characterization with BW water (BW pilot test, Section 3.1.1). The membranes with the lowest permeate fluxes and highest rejection were placed in the first positions as indicated in Table 5. From November 2019 until February 2022 (27 months), at least 25% of the system was operating with second-hand membranes. 

#### 3.3.1. Membrane Performance

**Figure 4:**
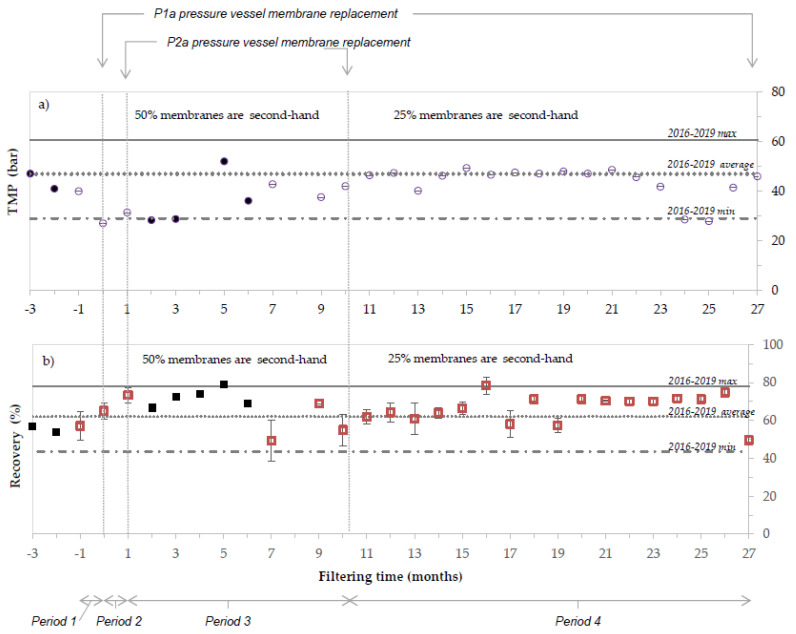
TMP (**a**) and recovery percentage (**b**) comparison between the period operating with second-hand membranes (monthly dots and squares, respectively) and the maximum, average and minimum values detected during 4 years of operation (in horizontal lines) with standard RO membranes (2016–2019).

Figure 4 shows the TMP (violet) and the recovery percentage (red) of the period operating with second-hand membranes (monthly average); the three vertical lines correspond to membrane replacements. Values plotted in negative months correspond to standard operation before any replacement (Figure 4, Period 1). In month 0 (Figure 4 Period 2), standard membranes of P1a pressure vessels were substituted by second-hand membranes, while those corresponding to P2a were replaced in month 1 (Figure 4, Period 3). In month 10, the regenerated membranes placed in P2a pressure vessel were substituted by standard membranes (Figure 4, Period 4), due to a failure of the last membrane (last position). Data collected by the technician were used when data from data logger was not available and are represented in black colour. Maximum, average and minimum values observed during 4 years of operation with standard RO membranes (2016–2019) are represented by horizontal lines. 

As observed in Figure 4, the usage of second-hand membranes did not impact negatively the performance of the process. During 9 months of operation (Period 3: from month 1 to month 10), the system was filtering water with 6 second-hand membranes (3 regenerated and 3 end-of-life, representing 50% of the total RO membranes). In such conditions, on average, the process operated at higher water production (historic data 2016–2019: 62 ± 9 % vs. 71 ± 4% recovery when using second-hand membranes) and at lower TMP (and energy) (historic data 2016–2019: 47 ± 5 bar TMP vs. 35 ± 8 bar TMP when using second-hand membranes). Two main factors might have contributed to this effect. On one hand the variable feed water quality; the average feed conductivity from 2016–2019 was 36.15 ± 3.72 mS/cm, while it dropped to 24.4 ± 3.8 mS/cm in the following year. On the other hand, the replacement of fouled membranes by clean membranes can also have an impact (according to the operator, the second-hand membranes behaved as if the membranes were new). 

After 10 months of operation, the last membrane module of the pressure vessel P2a broke, and the regenerated membranes were replaced by a standard membrane module. No significate changes were observed in terms of recovery or TMP. From months 11 to 26, the system operated with 25% second-hand membranes installed. The facility was operating with a similar TMP to the average of the historic data (historic data 2016–2019: 47 ± 5 bar TMP vs. 44 ± 6 bar TMP), while producing a higher volume of clean water (historic data 2016–2019: 62 ± 9 % vs. 67 ± 6 %). Month 27 was the last before the membrane replacement of pressure tube P1a.

#### 3.3.2. Statistical Results

**Figure 5:**
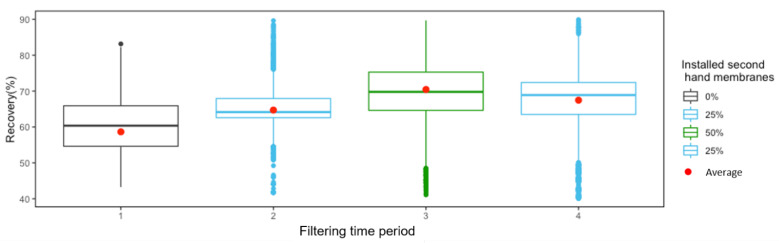
Boxplot corresponding to the 4 filtering periods of times showed in Figure 4 where the system was operating with standard RO membranes (period 1), 25% of second-hand membranes (periods 2 and 4) and 50% of second-hand membranes (period 3).

The % recovery was statistically assessed during the four operating periods (Figure 5). The median and average recovery are different between the periods, and both are higher when using second-hand membranes. Additionally, the highest values were achieved when 50% of the installed membranes were second-hand membranes. 

Kolmogorov-Smirnov test revealed the non-normality of the data in none of the periods, reaching a *p*-value lower than 0.05. Additionally, the variances of period 1 (using only standard RO membranes) were also compared with the rest of the periods (using second-hand membranes) conducting F-test. None of the periods are statistically similar to period 1, showing in all the cases a *p*-value < 1.4 e^−12^. Based on these results (non-normality and non-similar variances), a non-parametric test (Wikcox test) was used to compare the mean values. Results showed that there are significant differences between the mean values over each period (*p*-value < 2 e^−16^).

#### 3.3.3. Permeate Water Quality Control

The average and standard deviation of permeate water quality parameters related to organic matter (DQO, DBO5, TOC), ions and nitrogen compounds (N-NH_4_^+^, N-NO_2_^−^ and N-NO_3_^−2^) for the 4 pressure vessels of the RO system are presented in Table 7. Green and red colours indicate that the second-hand membranes were performing respectively better and worse than the standard RO membranes. The permeate water analyses of P1a pressure vessel (end-of-life RO membranes) are comparable to those of P1b pressure vessel (standard membranes) and those of P2a pressure vessel (regenerated membranes) are comparable to those of P2b pressure vessel (standard membranes). 

Overall, second-hand membranes gave similar performances to commercial ones. The standard deviation shows a great variability in the water quality. In most of the cases, the average value achieved when using second-hand membranes was within the margin of error. The largest variations were observed for DBO_5_, Cl^−^ and Na^+^. Although, the water quality was within the limits to be safely discarded [36], water was internally reused in the landfill facility. Note that values shown in Table 7 corresponded to the permeate of single pressure tubes, not the total permeate produced in the plant.

Water sampling allowed to determine membrane failure in the 10^th^ month (September 2020, punctual analysis shown in Table 8)). The permeate of P2a pressure vessel showed a yellowish colour and all the water quality analysis where worse than the standard SWRO membranes (P2b). The colour could be due to the increment of organic matter passage, confirmed by the increase in COD, TOC and SO_4_^−2^ values in P2a, which were 20% higher than for P2b. The second-hand membrane placed in position 3 of pressure vessel P2a showed external physical damage (end-cap detached from the rest of the module). The second-hand membrane placed in position 3 of pressure vessel P2a showed external physical damage (end-cap detached from the rest of the module). In order to minimize the risk of failure in their second life a non-invasive inspection should be performed, ensuring the physical integrity of the external casing and the end-caps.

### 3.4. Membrane Autopsy

Membrane autopsy was conducted through visual and analytic assessment. Three membranes of LGSW440GR model were dismantled. 

**Figure 6:**
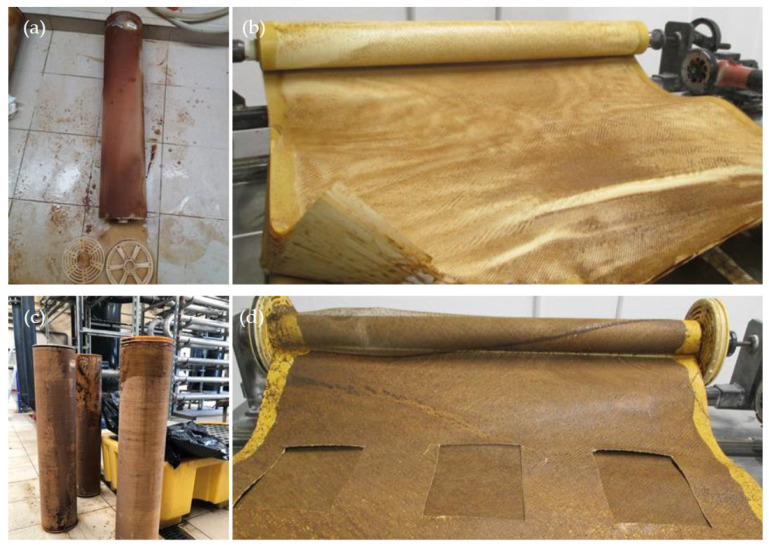
M4 (**a**,**b**) regenerated membrane after treating LFL for 10 months. M5 (**c**,**d**) end-of-life membrane directly reused for 27 months.

As shown in Figure 6, severe fouling was observed, distributed both outside the fiberglass shell and inside the membrane modules. The M4 and M5 membranes were treated LFL during 10 and 27 months, respectively, at the LFL industrial facility. After dismantling the membrane module, coupons and fouling samples were collected for analysis. Dry membrane fouling weight, TGA and IPC analysis are shown in Table 8. Membrane fouling was more significant in the M5 membrane sample than in M4 one, consistently with the longer operating time. TGA showed that in both cases, most of the fouling was inorganic. ICP analysis revealed that the major common inorganic compounds were: Na, K, Mg, Ca, S, Fe, Al, Sn, Ti.

**Figure 7:**
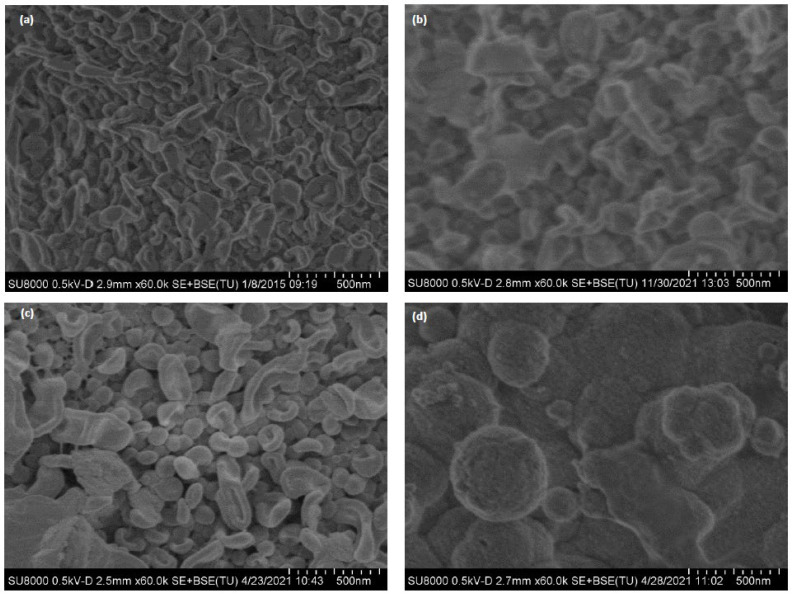
SEM images of (**a**) non-used PA RO membrane (TM720–400, Toray), (**b**) M1 end-of-life RO membrane; (**c**) M1 regenerated RO at laboratory scale (**d**) fouled-regenerated membrane (M4) after having been treating LFL during 10 months.

Figure 7 shows SEM images of membrane surface for a non-used (Figure 7a), end-of-life RO membrane (M1) (Figure 7b), regenerated RO membrane at laboratory scale (M1) (Figure 7c) and regenerated RO membrane at pilot scale (M4) after operating in the full-scale LFL facility for 10 months (Figure 7d). As expected, the morphology of the M1, end-of-life and regenerated membrane coupons was similar to the RO pristine surface, indicating that end-of-life RO membrane from the desalination facility did not present high level of fouling. The morphology of the M4 regenerated but fouled membrane was quite different, as fouling covered the main surface. SEM of the M5 membrane sample was not conducted due to the high level of fouling identified in the autopsy.

**Figure 8:**
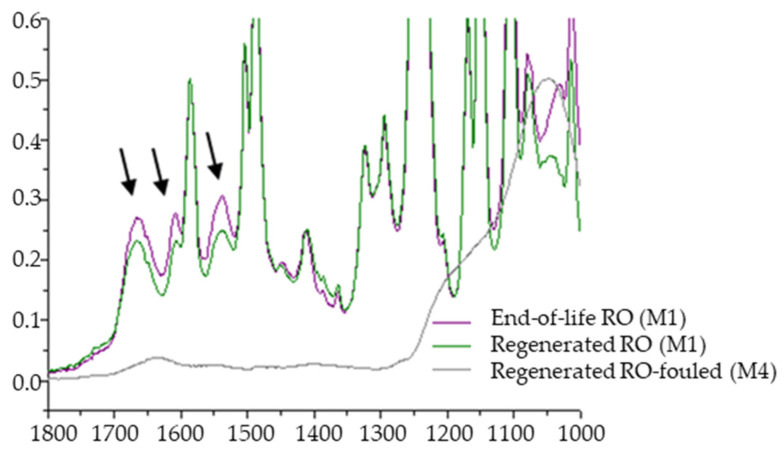
ATR–FTIR spectra for LGSW440 GR membrane end-of-life RO membrane: M1 end-of-life, M1 regenerated and M4 regenerated but fouled after treating LFL (peaks indicated with arrows correspond to the PA signals).

On the other hand, the low degradation of the PA layer was investigated by the ATR–FTIR spectroscopy (Figure 8). The spectra of the M1 membrane surface before and after the regeneration treatment shows that both end-of-life and regenerated membranes had peaks at 1664 cm^−1^, 1542 cm^−1^ and 1610 cm^−1^. These peaks correspond to amide I and amide II bands and the C = C stretching vibrations from the aromatic amide bonds, respectively [37]. Nonetheless, the peaks signals were barely reduced in case of the regenerated membranes, which indicates the presence of the PA layer. 

The spectrum of the M4 membrane did not show those typical peaks of PA layer, due to fouling present on its surface. 

## 4. Conclusions

End-of-life RO membrane management still follows an economical linear model. Generally, membranes are used in a single process and afterwards, disposed in landfill without considering other potential uses. This work shows that direct reuse and regenerated RO membranes reuse are a suitable alternative management to extend the SWRO membranes lifespan. 

Membrane characterization at pilot scale using BW revealed that a proper sorting is advised to avoid membrane performance variability during their second life. Indeed, all the membranes exposed to the regenerating solution increased their original flux without compromising their rejection capability. 

Short-term experiments at laboratory scale demonstrated that the end-of-life RO membrane model LG SW440GR (LG) was suitable for being reused in LFL treatment. and then validated in long-term experiments at full scale LFL facility, operating in the standard conditions for up to 27 months. Both end-of-life and regenerated RO membrane performed similar to the commercial RO membranes already used in the facility. Permeate colour as well as COD, TOC and SO_4_^−2^ passage could potentially be used to detect membrane failure.

Normally, new RO membranes treating LFL last from 12 to 36 months. This work demonstrated a reasonable second life (from 10 to 27 months) for RO membranes discarded in the SW industry. Therefore, it is expected that the LFL treatment industry will be interested in reusing second-hand membranes, introducing a circular economy practice in their process. Second-hand membranes should be validated in other high-rate replacement processes, proving other potential segments of the wide range of RO applications.

## Figures and Tables

**Table 1:** Information of the end-of-life RO membranes used in this study.

Internal Code	Membrane Brand and Model	Physical External Damage at the End of the First Life	Sorting (BW Pilot Test)	Regeneration (Lab * and Pilot ** Scale)	Reuse Test. LFL Lab Scale	Reuse Test. LFL in Industry	Autopsy
M1	LG Chem’s LGSW440GR (41 m^2^)	X	X	X *	X		X
M2			X	X **		X	
M3			X	X **			
M4			X	X **		X	X
M5			X			X	X
M6			X			X	
M7			X			X	
M8			X			X	
M9			X				
M10	TORAY TM820M 400 (37 m^2^)	X	X	X **	X		X
M11			X				
M12			X	X **			

**Table 2:** Operating conditions and BW quality.

Pressure (Bar)	Temperature (°C)	pH	Conductivity (mS/cm)	Cl^−^ (ppm)	N-NO_3_^−^ (ppm)	SO_4_^2−^ (ppm)
15.5	23 ± 1	7.4 ± 0.1	24,076 ± 536	7570.0	152.0	3186.0

**Table 3:** Water quality parameters of a feed LFL sample.

Ph		Conductivity (µs/cm)				DQO (ppm)	DBO_5_ (ppm)	TOC (ppm)	TC (ppm)	IC (ppm)
7.9		38,600				3660	300	1198	1990	791
**F^−^** **(ppm)**	**Cl^−^** **(ppm)**	**N-NO_2_^−^** **(ppm)**	**N-NO_3_^−^** **(ppm)**	**PO_4_^−3^** **(ppm)**	**SO_4_^−2^** **(ppm)**	**Na^+^** **(ppm)**	**N-NH_4_^+^** **(ppm)**	**K^+^** **(ppm)**	**Ca^2+^** **(ppm)**	**Mg^2+^** **(ppm)**
3.5	10,586.0	8.7	189.0	20.7	1897.0	7427.0	0.61	2956	101.0	233.0

**Table 4:** Ultrafiltered LFL quality for the period of 01–2016 to 12–2020 used as RO feed.

Parameter	Feed Average Concentration (ppm)
COD	3448.4 ± 527.4
Conductivity (µs/cm)	32,800 ± 5200
Cl^−^	8460.5 ± 1398.1
N-NO_3^−^_	84.4 ± 135.4

**Table 5:** Type of second-hand membranes installed in two pressure vessels of an LFL full-scale facility.

Date (Initial-End)	Pressure Vessel	Type of Second-Hand Membranes	Membranes Placed at the Position: 1, 2, 3
7 November 2019–10 February 2022	P1a (down)	End-of-life RO	M5, M7, M8
3 December 2019–1 October 2020	P2a (up)	End-of-life and Regenerated RO *	M6, M2 *, M4 *

*: regenerated.

**Table 6:** Permeate flux and rejection coefficients for standard RO membranes used in the LFL industrial facility compared to end-of-life RO membrane (M1), and regenerated membranes (M1, M10).

Membrane	Standard RO Used in the Industrial Facility (2016–2019) *	M1 RO- End-of-Life	M1 Regenerated RO	M10 Regenerated RO
**MQ water permeate flux** **(L h^−1^ m^−2^)**	NA	72.27 ± 6.97 (45 bar)	115.03 ± 7.41 (45 bar)	201.36 ±15.38 (45 bar)
**Landfill leachate permeate flux (L h^−1^ m^−2^)**	1.51 ± 0.25 (47 ± 5 bar)	32.66 ± 0.62 (45 bar)	46.38 ± 0.45 (45 bar)	64.29 ± 1.04 (45 bar)
**Landfill leachate permeability (L h^−1^ m^−2^ bar^−1^)**	0.03 ± 0.01	0.73 ± 0.01	1.03 ± 0.01	1.43 ± 0.02
**Rejection coefficients (%)**				
**Conductivity**	95.5 ± 2.0	98.9	98.6	98.1
**COD**	97.9 ± 0.6	99.6	99.5	99.8
**BOD5**	95.8 **	NA	98.0	NA
**TOC**	NA	99.4	99.3	99.8
**TC**	NA	99.3	99.2	99.3
**IC**	NA	99.0	99.0	98.7
**F^−^**	NA	99.1	98.8	99.1
**Cl^−^**	95.7± 3.2	99.1	98.9	98.4
**N-NO_2_^−^**	N.A	98.3	97.8	93.4
**N-NO_3_^−2^**	84.1 ± 5.1	98.2	97.6	92.3
**PO_4_^−3^**	NA	97.6	97.6	97.6
**SO_4_^−2^**	99.2 **	99.5	99.4	99.8
**Na^+^**	NA	99.2	99.0	98.6
**N-NH_4_^+^**	58.7 ± 15.9	NA	97.9	98.4
**K^+^**	NA	99.1	99.0	98.8
**Ca^+ 2^**	NA	99.4	99.4	99.8
**Mg^+2^**	NA	99.4	99.5	99.9
**Color**	No	No	No	Yes

* Data generously facilitate by TELWESA company for 2016–2019 period. ** Data from a punctual sample (July 2019). NA, Data not available.

**Table 7:** Comparison of RO permeate quality of the fourth pressure vessel during long-term operation: P1a-with SWRO non-treated RO membranes, P1-b with standard SWRO membranes, P2-a with SWRO regenerated membranes, and P2-b with standard SWRO membranes.

Parameter	Discharge Water Quality Limits According to Spanish Law [36]	Month 0–Month 27 (12 Water Samples)						Month 1–Month 10 (6 Water Samples)						Month 10 (08 September 2020) Punctual Analysis	
		Second-Hand SWRO-(P1a) End-of-Life			Standard SWRO (P1b)			Second-Hand SWRO-(P2a) Regenerated			Standard SWRO (P2b)			Second-Hand SWRO-(P2a) Regenerated	Standard SWRO (P2b)
**pH**	6–9	5	±	1	6	±	1	6	±	1	6	±	1	N.A	N.A
**Conductivity** **(µS/cm)**	5000	524	±	221	680	±	338	2795	±	852	2852	±	948	N.A	N.A
**COD** **(ppm)**	1600	65	±	65	67	±	66	132	±	130	133	±	113	**384**	301
**DBO_5_** **(ppm)**	1000	168	±	120	145	±	107	196	±	87	177	±	83	205	197
**TOC** **(ppm)**	N.A	9	±	19	11	±	21	63	±	55	57	±	78	**146**	121
**N-NH_4_** **(ppm)**	60	17	±	22	21	±	29	51	±	61	52	±	51	<D.V	<D.V
**N-NO_2_** **(ppm)**	N.A	17	±	39	15	±	34	<D.V			<D.V			<D.V	<D.V
**N-NO_3_** **(ppm)**	50	5	±	14	7	±	16	3	±	4	2	±	3	9	8
**Cl-** **(ppm)**	N.A	127	±	148	110	±	92	724	±	413	672	±	380	1519	1357
**SO_4_** **(ppm)**	N.A	2	±	3	2	±	2	45	±	50	40	±	45	120	100
**Na** **(ppm)**	N.A	56	±	24	70	±	21	627	±	264	575	±	244	1101	981
**K** **(ppm)**	N.A	38	±	19	40	±	15	269	±	99	254	±	86	447	399

N.A. = Data not available. D.V: detectable value.

**Table 8:** Dry membrane fouling weight, TGA and ICP result analysis for two membranes that were operating under industrial conditions in an LFL treatment facility.

Internal Code	Membrane Description	Dry Membrane Fouling Eeight (g·cm^−2^)	TGA	ICP Analysis (g/kg)
M4	Regenerated membrane after 10 months treating LFL. Placed in the last position of the second step (P2a pressure vessel)	7.0	Inorganic: 67.0% Organic: 33.0%	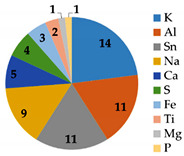
M5	End-of-life membrane (directly reused) after 27 months treating LFL. Placed in the first position of the first step (P1a pressure vessel)	37.0	Inorganic: 59.5% Organic: 40.5%	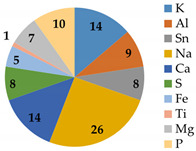

## Data Availability

Not applicable.

## Nomenclature

ATR-FTIR	attenuated total reflectance-Fourier transform infrared
BW	brackish water
BOD5	5 days biochemical oxygen demand
COD	chemical oxygen demand
IC	ion chromatography
ICP-MS	inductively coupled plasma mass spectrometry
LFL	landfill leachate
NF	nanofiltration
PA	polyamide
RO	reverse osmosis
UF	ultrafiltration
TGA	thermogravimetric analysis
TMP	transmembrane pressure
TOC	total organic carbon
SEM	Scanning electron microscopy
SW	seawater
SWRO	seawater reverse osmosis membranes
